# Long-Term Detection of Glycemic Glucose/Hypoglycemia by Microfluidic Sweat Monitoring Patch

**DOI:** 10.3390/bios14060294

**Published:** 2024-06-05

**Authors:** Wenjie Xu, Lei Lu, Yuxin He, Lin Cheng, Aiping Liu

**Affiliations:** Key Laboratory of Optical Field Manipulation of Zhejiang Province, School of Science, Zhejiang Sci-Tech University, Hangzhou 310018, China; 202120103111@mails.zstu.edu.cn (W.X.); 202230106309@mails.zstu.edu.cn (L.L.); hyx980662@163.com (Y.H.)

**Keywords:** microfluidics, sweat patch, glycemic/hypoglycemic, long-term monitoring

## Abstract

A microfluidic sweat monitoring patch that collects human sweat for a long time is designed to achieve the effect of detecting the rise and fall of human sweat glucose over a long period of time by increasing the use time of a single patch. Five collection pools, four serpentine channels, and two different valves are provided. Among them, the three-dimensional valve has a large burst pressure as a balance between the internal and external air pressures of the patch. The bursting pressure of the two-dimensional diverter valve is smaller than that of the three-dimensional gas valve, and its role is to control the flow direction of the liquid. Through plasma hydrophilic treatment of different durations, the optimal hydrophilic duration is obtained. The embedded chromogenic disc detects the sweat glucose value at two adjacent time intervals and compares the information of the human body to increase or reduce glucose. The patch has good flexibility and can fit well with human skin, and because polydimethylsiloxane (PDMS) has good light transmission, it reduces the measurement error caused by the color-taking process and makes the detection results more accurate.

## 1. Introduction

Sweat can convey significant health information, as both the rate and volume of sweat secretion can reflect various health states. Sweat detection and analysis are anticipated to supplant blood tests for puncture injuries, potentially reducing the risk of wound infections [1,2,3]. Research has demonstrated that sharp fluctuations in blood sugar levels pose serious potential harm to human health. Fluctuations in blood sugar can induce abnormal activation of the sympathetic nervous system, leading to increased morbidity and mortality from cardiovascular diseases [4,5]. Moreover, excessive blood glucose fluctuations can lead to oxidative stress, endothelial damage, and the activation of inflammatory processes and coagulation mechanisms, among other conditions [6]. In cases of excessive blood sugar fluctuation, immediate hospitalization or administration of hypoglycemic drugs as per medical advice is necessary to prevent more serious conditions. Consequently, monitoring blood sugar levels is crucial for mitigating the risk of emergent diseases, real-time health monitoring, and ensuring the timely administration of medications [7].

The capabilities of microfluidic controllers for real-time feedback detection offer promising research prospects in monitoring fluctuations in human sweat sugar levels. For instance, Martin et al. employed screen-printing techniques to integrate a small flexible circuit board with a microfluidic collection patch for sweat detection, succeeding in quickly channeling the collected sweat into an electrochemical sensing chamber for glucose and lactate detection, albeit with a relatively brief detection duration [8]. Xiao et al. developed a glucose colorimetric sensor equipped with a check valve to address skin contamination issues due to water evaporation from sweat samples and the backflow of detected chemical substances, also enabling the collection of five sweat samples simultaneously. The randomness and error in detection are mitigated through five parallel measurements, though the mixing of old and new sweat samples could pose a challenge [9]. Furthermore, given the requirement for microfluidic devices to be conveniently wearable on the skin, their typically small size complicates the continuous monitoring of sweat sugar fluctuations over extended periods. The frequent replacement of detection patches can escalate costs and hinder routine real-time monitoring. Consequently, the brief detection intervals and the absence of a pronounced hypoglycemic index over short durations have emerged as challenges in the dynamic monitoring of sweat sensors [10,11]. These issues underscore the need for further advancements in microfluidic technology to enable efficient, cost-effective, and long-term monitoring of health indicators through sweat analysis.

Herein, we describe a microfluidic sweat monitoring patch that can collect and analyze human sweat over extended periods. The patch comprises an encapsulation layer of cured PDMS, a sensing layer made from a laser-cut dust-free paper disc, and a microchannel layer formed by replicating a 3D-printed mold of PDMS. Within the microchannel layer, there are five collection cells, four serpentine channels, and both two-dimensional (2D) and three-dimensional (3D) valves. The 3D gas valve functions as an air release mechanism, retaining sweat due to its high burst pressure, while the 2D diverter valve, with a lower burst pressure, controls the liquid flow. PDMS’s superior light transmittance renders it ideal as the primary material for the colorimetric sensing patch, reducing errors in colorimetric analysis and enhancing the accuracy of the detection results. Capable of detecting fluctuations in human sweat sugar levels over prolonged periods, this patch is distinguished as having the longest known detection time among microfluidic sweat sugar monitoring devices. This innovation opens new avenues for non-invasive, long-term monitoring of health indicators through sweat analysis.

## 2. Design and Preparation of Microfluidic Sweat Glucose Monitoring Patch

### 2.1. Preparation of Microfluidic Sweat Glucose Monitoring Patches

**Figure 1:**
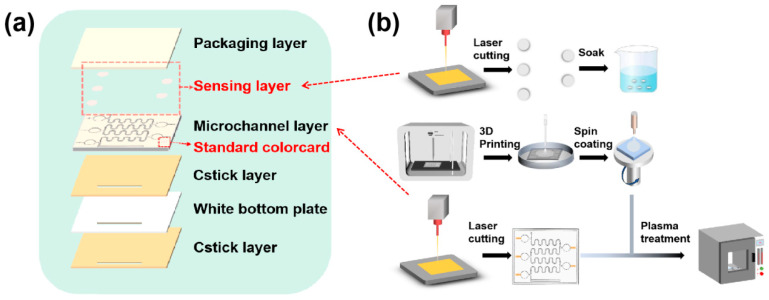
(**a**) Microfluidic-controlled sweat sugars to monitor the overall construction of the patch. (**b**) Flow chart of the microfluidic-controlled sweat sugar monitoring patch.

The microfluidic sweat patch is composed of six layers arranged from top to bottom: the encapsulation layer, sensing layer, microchannel layer, adhesive layer I, white backing, and adhesive layer II (Figure 1a). The fabrication process of the sensing layer is illustrated in Figure 1b. Initially, dust-free paper is laser-cut into round discs with a 2.8 mm radius to ensure they fit within the collection cell while leaving an air venting gap to prevent obstruction of the air venting channel. Subsequently, the cut discs were immersed in a Glucose Oxidase-Peroxidase (GOD-POD) working solution at 4 °C for 3 h, then removed and naturally dried to produce the color development discs for the sensing layer. The GOD-POD working solution, a 1:1 mixture of phenol and enzyme reagents, is thoroughly mixed after adding an equal amount of glucose oxidase. The microchannel layer consisted of a PDMS sheet with microstructured grooves, including a sweat collection inlet (2 cm × 1 mm), a serpentine channel, a 2D diverter valve, and a 3D gas valve (Figure 1b). Sweat, secreted by the body’s sweat glands, enters the patch via the collection inlet, with the original air within the microfluidic channels expelled through the 3D gas valve. Initially, sweat fills the first collection pool and reacts with the color-developing discs in the sensing layer, producing a color change that indicates the initial sweat sugar concentration. As sweat secretion continues, the liquid pressure within the channels increases, eventually exceeding the burst pressure of the 2D diverter valve. This pressure directs the flow through the manifold valve into the serpentine channel, filling it and leading into the second collection pool to obtain subsequent sweat sugar values. This process repeats until all five collection pools are filled, tracking changes in sweat sugar over time, and providing a detailed analysis of sweat sugar fluctuations.

The white bottom plate, made from flexible PVC, serves as a background to minimize the color variance across different skin tones. This ensures the color accuracy of the sensing layer’s color-developing discs and reduces errors in color interpretation. Both adhesive layer I and adhesive layer II are fashioned from double-sided adhesive tape, precisely shaped through laser cutting. Adhesive layer I functions to securely bond the microchannel layer to the white substrate, while adhesive layer II, the bottommost layer of the microfluidic sweat monitoring patch, facilitates easy attachment to the skin’s surface for wearer comfort. Following an ionophilic hydrophilic treatment, the encapsulation layer of cured PDMS can be fully bonded to the microchannel layer, marking the completion of the encapsulation process. The overall thickness of the patch is 2.24 mm.

### 2.2. Design of Three-Dimensional Gas Valves

In order to achieve sequential collection, it is critical to design the air and diverter valves [12]. In this regard, we carried out a theoretical analysis based on 3D gas valves and 2D diverter valves. In the microfluidic sweat sugar monitoring patch, when the liquid flows into the microchannel structure, the channel can be approximated as a rectangular capillary, and the front surface of the liquid can be driven forward into a convex spherical shape [13]. The driving force, which is the internal and external pressure difference of the spherical liquid surface Δ*P*, meets the Yang equation [14].

(1) $$∆P_{1}=P_{A}-P_{O}=-\sigma \left(\frac{\cos\theta_{1}+\cos\theta_{2}}{w}+\frac{\cos\theta_{3}+\cos\theta_{4}}{h}\right),$$

where *P_A_* and *P_O_* are the capillary pressure inside and outside the surface, *σ* is the surface tension coefficient of the liquid, *w* and *h* are the width and height of the channel respectively, and *θ*_1_, *θ*_2_, *θ*_3_ and *θ*_4_ are the contact angles between the liquid and the upper, lower, left, and right contact surfaces of the channel.

When the liquid moves from the flat channel to the valve, the channel suddenly widens, the liquid flow path shows a divergent trend, and the three-phase contact line of the liquid immediately stops moving at the divergent valve. In order to pass through the valve, the contact angle between the front surface of the liquid and the inner wall of the channel is increased. When the contact angle increases to a certain angle, the liquid continues to advance through the blasting valve [15]. When the liquid is still in the microcirculation channel, the flow state of the liquid will produce distinct differences from the macroscopic fluid motion. Due to the surface tension of the liquid, the liquid is in the wetting phase. The pressure difference inside and outside the front surface of the liquid level satisfies Young’s equation.

For the uniform patch of the encapsulation layer in the same material as the microchannel layer, *θ*_1_ *= θ*_2_ *= θ*_3_ *= θ*_4_ *= θ_v_* in the valve-free pass-through, where *θ_v_* is the water contact angle on the PDMS surface. In the rectangular direct channel, the internal and external pressure difference of the spherical liquid surface Δ*P*_1_ is [16]

(2) $$∆P_{1}=P_{A}-P_{O}=-\sigma \left(\frac{2\cos\theta_{v}}{w}+\frac{2\cos\theta_{v}}{h}\right)=-2\cos\theta_{v}\sigma /R,$$

where *R* = *wh*/*w* + *h*, is the hydraulic radius of the rectangular straight channel.

Since PDMS itself is hydrophobic, the water contact angle on the surface of PDMS can be known, *θ_v_* ∈ (90°, 180°).

(3) $$∆P_{1}=2\left|\cos\theta_{v}\right|\sigma /R,$$

**Figure 2:**
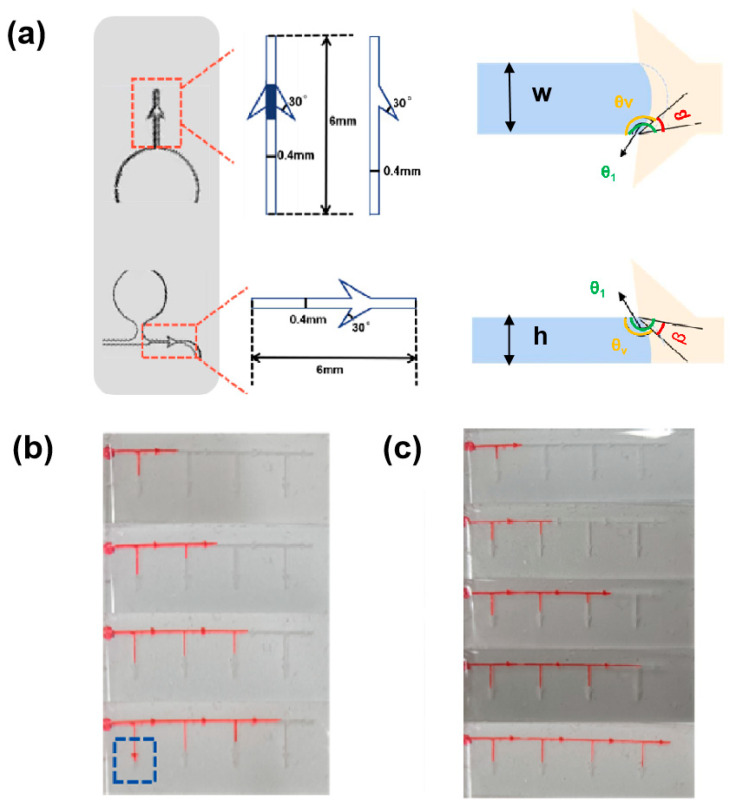
(**a**) Schematic of the structure of a 3D gas valve and a 2D diverter valve. (**b**) Liquid flow state when the secondary valve is set as a 2D diverter valve. (**c**) Liquid flow state when the secondary valve is set as a 3D air valve.

When the valve is a 2D flat valve, as shown in Figure 2, the liquid flows to the valve inlet of the microchannel, because the channel *w* becomes wider and *h* does not change, so the channel diverges to the left and right sides, and the contact angle of both sides becomes larger. The pressure difference between the inside and outside of the liquid spherical liquid level, that is, the valve burst pressure, is

(4) $$∆P_{2}=P_{A}-P_{O}=-2\sigma \left(\frac{\cos\theta_{v+\beta }}{w}+\frac{\cos\theta_{v}}{h}\right)=-2\sigma \left(\frac{\cos\theta_{I}}{w}+\frac{\cos\theta_{v}}{h}\right),$$

Among them, the *β* is the diffusion angle generated by the left and right contact surfaces at the sudden change in the size of the channel. *θ_I_* ∈ (90°, 180°].

When cos*θ_I_* = 180°, the liquid passes through the burst valve, at this time

(5) $$∆P_{2}=-2\sigma \left(-\frac{1}{w}+\frac{\cos\theta_{v}}{h}\right)=\frac{2\sigma }{w}+\frac{2\sigma \left|\cos\theta_{v}\right|}{h},$$

When the channel size is fixed, when the contact angle becomes larger, the burst pressure required for the liquid to break through the valve increases.

When the valve is a 3D gas valve, when the liquid flows to the valve, the upper, left, and right channels of the channel spread, and its top view is the same as the 2D valve, and the front view is shown in Figure 2.

The pressure difference between the inside and outside of the spherical liquid level of the channel at the valve inlet is

(6) $$∆P_{3}=P_{A}-P_{O}=-\sigma \left(\frac{2\cos\theta_{I}}{w}+\frac{\cos\theta_{v}+\cos\theta_{I}}{h}\right),$$

burst pressure required for the liquid to pass through the valve is

(7) $$∆P_{3}=-\sigma \left(-\frac{2}{w}+\frac{\cos\theta_{v}-1}{h}\right)=\frac{\left(w+2h\right)\sigma }{wh}+\frac{\sigma \left|\cos\theta_{v}\right|}{h},$$

Compare the pressure required to pass a 2D flat burst valve and a 3D stereo burst valve

(8) $$∆P^{\prime }=∆P_{3}-∆P_{2}=\frac{\left(w+2h\right)\sigma }{wh}+\frac{\sigma \left|\cos\theta_{v}\right|}{h}-\left(\frac{2\sigma }{w}+\frac{2\sigma \left|\cos\theta_{v}\right|}{h}\right),$$

(9) $$∆P^{\prime }=\frac{\sigma }{h}\left(1-\left|\cos\theta_{v}\right|\right),$$

(10) $$\left|\cos\theta_{v}\right|\in \left(0,1\right),$$

Specifically, the bursting pressure for the 3D valve exceeds that of the 2D valve. Consequently, a 3D valve, requiring higher bursting pressure, is utilized as the gas valve, while a 2D valve, necessitating lower bursting pressure, serves as the flow control valve. Once a collection tank is filled, the liquid is directed into the serpentine channel via the 2D diverter valve, rather than overflowing through the 3D valve. This design ensures the liquid sequentially fills the five collection tanks along a predetermined path, preventing leakage. This operational mechanism highlights the sophisticated design of the microfluidic sweat patch, enabling precise control over fluid dynamics to ensure efficient and accurate sweat collection and analysis. 

Further experiments were carried out to verify that the liquid could pass through the 2D diverter valve without any problems, while the burst pressure was less than that of the 3D gas valve [17]. The tests were performed by simulating an injection with a syringe pump. A 2D diverter valve was set up as the primary valve in a straight-through channel with a width of 0.6 mm, and a branch channel was introduced on one side of the straight-through channel, which also had a 2D diverter valve as the primary valve. The secondary valve is located on the branch channel, which is equal to the width of the valve inlet. Red dye was injected from the channel side with a syringe pump at an injection rate of 1 μL/min. Since the straight-through channel was slightly wider than the branch channel, the liquid flowed preferentially into the main channel valves. When the liquid flowed through the third set of valves, the liquid in the straight-through channel passed through the secondary valves on the branch channel (Figure 2b), indicating that at this time the liquid pressure was already greater than the burst pressure of the 2D diverter valve, and the liquid in the channel overflowed [18]. The secondary valve on the branch channel was replaced with a 3D gas valve (Figure 2c), and the injection experiment was repeated, and the liquid passed through four sets of valves without liquid leakage, indicating that the liquid pressure was always less than the burst pressure of the 3D gas valve. 

### 2.3. Hydrophilic Treatment of the Inner Wall of Microfluidic Channels

**Figure 3:**
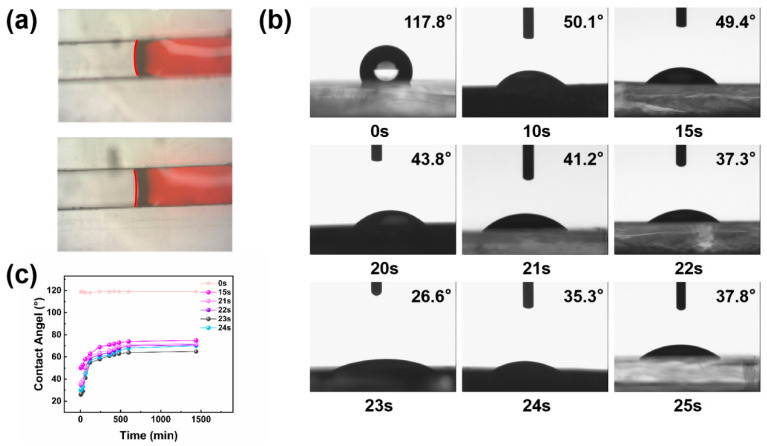
(**a**) Shape of the liquid’s leading surface in hydrophobic and hydrophilic channels. (**b**) Water contact angles of PDMS with different hydrophilic treatment duration. (**c**) Contact angles of PDMS with different hydrophilic time at 5 min, 10 min, 30 min, 1 h, 2 h, 4 h, 6 h, 7 h, 8 h, 10 h, and 24 h.

In channels made of hydrophobic materials, the precursor liquid forms a convex spherical surface, necessitating a higher liquid pressure to overcome the channel resistance and propel the flow, with new liquid pushing forward the old. Conversely, in channels made of hydrophilic materials, the precursor liquid forms a concave spherical surface (Figure 3a). Capillary action allows the liquid to advance along the channel’s inner wall, enabling autonomous flow without external propulsion. To maximize sweat collection time and extend the interval for detecting sweat sugar changes, the channel’s inner wall underwent hydrophilic treatment via plasma. Initially, cured PDMS sheets were cleansed with an ethanol solution to remove surface impurities before being placed in the plasma cleaner’s inner chamber for batch processing at set intervals: 10 s, 15 s, 20 s, up to 25 s. After cleaning, a drop of deionized water was applied to the PDMS sheet’s surface using a rubber-tipped buret. The droplet’s shape was recorded, and its contact angle was measured with a DCA-322 dynamic contact angle analyzer [19]. This meticulous approach ensures that the channels within the microfluidic sweat patch are optimally prepared for efficient sweat collection and analysis, highlighting the importance of surface treatment in the functionality of microfluidic devices. The findings are presented in Figure 3b. Experimental observations indicate that prior to 23 s, the contact angle of PDMS decreases as the hydrophilic treatment duration increases, reaching a minimum at 23 s. Beyond 23 s, PDMS hydrophilicity diminishes as treatment duration extends, resulting in a gradual increase in the contact angle. This establishes a strong correlation between the surface hydrophilicity of the material and the duration of hydrophilic treatment [20]. Within an optimal range, plasma treatment generates a significant number of free radicals and polar molecules on the surface, enhancing hydrophilicity to a dynamic maximum with increased treatment duration. However, beyond this optimal range, the saturation of free radicals and polar molecules occurs, leading to surface damage and reduced hydrophilicity with overly prolonged treatment [21]. 

Given that the hydrophilic properties of PDMS induced by plasma treatment are temporary, the contact angle gradually reverts to its original state over time [22]. We further investigated the hydrophilicity recovery in PDMS by examining flakes treated for 15 s, 21 s, 22 s, 23 s, 24 s, and untreated samples, measuring their water contact angles at specified intervals ranging from 5 min to 24 h. The experimental findings indicate that the water contact angle of hydrophilic-treated PDMS continuously increases for up to 7 h, then stabilizes between 7 and 8 h. Hydrophilic stability is somewhat preserved for 24 h, with the material’s hydrophobicity not fully reverting to its original state (Figure 3c). This exploration highlights the transient enhancement in hydrophilicity post-plasma treatment and its implications for the material’s use in time-sensitive applications. This exploration highlights the control of hydrophilic microchannels after plasma treatment on the flow rate of sweat through the microchannels.

**Figure 4:**
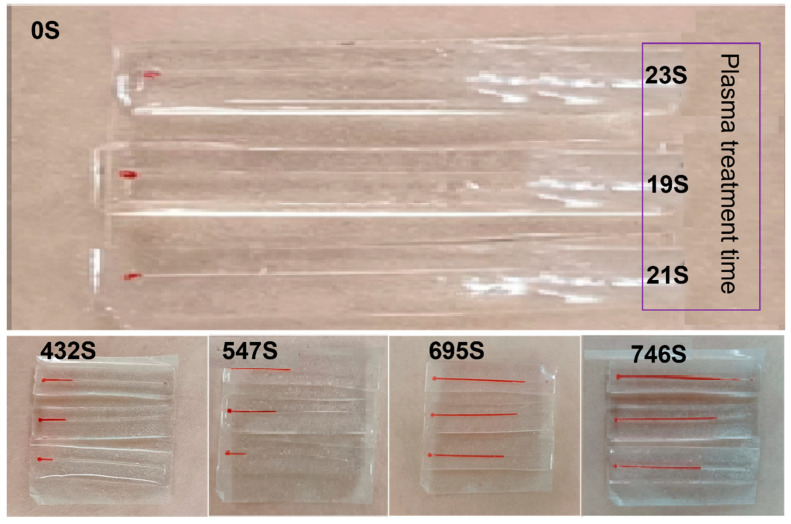
Comparison of the speed of sweat collection in straight-through plasma hydrophilic treatment 19 s, 21 s, 23 s.

To optimize the sweat flow duration through the microchannels while ensuring smooth entry, a comparative test was performed to ascertain the optimal hydrophilic treatment duration. Three straight microchannels underwent hydrophilic treatment for 19 s, 21 s, and 23 s, respectively (Figure 4). They were then encapsulated and left for 24 h to stabilize the hydrophilicity before testing. After 24 h, the microchannels were attached to the volunteers’ lower arms. For observation purposes, a small quantity of red dye powder was introduced into the collection cell. During exercise-induced sweating, the channel treated for 23 s was the first to fill with sweat, while the channel treated for 21 s took the longest to fill. Consequently, to regulate sweat flow rate and extend collection time, a treatment duration of 21 s was selected as the optimal hydrophilic treatment time for the microfluidic sweat collection patch. 

### 2.4. Microfluidic Channel Serpentine Design

**Figure 5:**
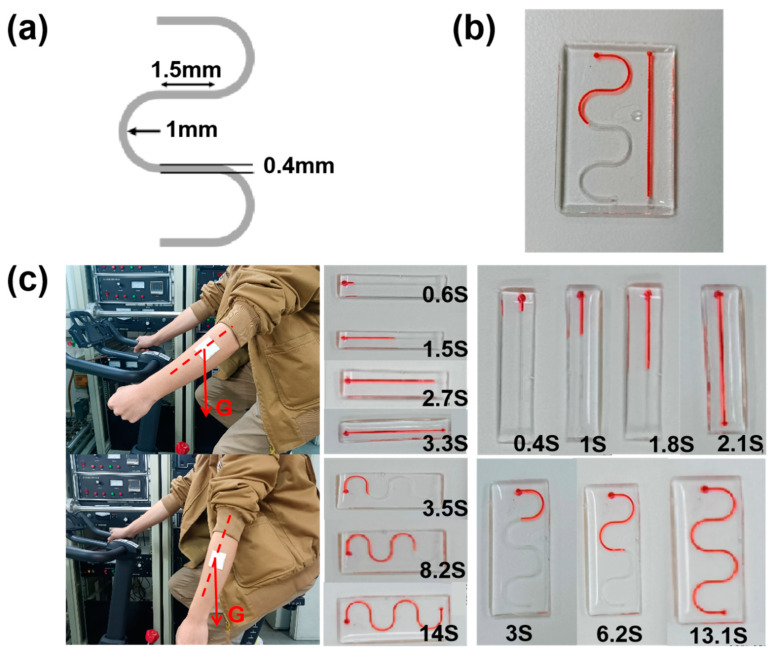
(**a**) Schematic structure of the serpentine channel. (**b**) Comparison diagram of the flow velocity between the serpentine channel and the straight channel. (**c**) Optical diagram of the change in the angle of the microfluidic patch with respect to gravity with the movement of the volunteer’s pendulum arm, and the collection time lengths of the through-channel and the serpentine channel when placed horizontally and vertically.

Acknowledging the influence of microchannel design on sweat flow timing, we implemented a serpentine design for the microchannels. As depicted in Figure 5a, the serpentine channel’s width was set at 0.4 mm, with a circular turn radius of 1 mm, a direct channel length of 1.5 mm between turning semicircles, and an internal channel height of 0.6 mm. For comparison, straight channels of equivalent linear length were fabricated. When injected at the same rate using a syringe pump, the serpentine channel’s flow time was approximately twice as long as that of the straight channel (Figure 5b). This indicates that the serpentine channel design effectively extends sweat collection time compared to the straight channel, showcasing the serpentine design’s advantage in prolonging the duration for analyzing sweat flow dynamics.

It is important to consider that the patch’s orientation relative to the body will vary during sweat collection based on the wearer’s movements, influencing the rate of sweat collection. When the fluid flow’s direction aligns with gravity (less than 90°), gravity accelerates the sweat flow, resulting in a faster flow and a shorter collection time. Conversely, when the liquid flow’s direction diverges from gravity (greater than 90°), gravity decelerates the sweat flow, slowing it down. In practical applications, minimizing gravity’s impact on sweat flow is crucial to ensure consistent intervals between measurements. In our experiments, the direct channel was oriented horizontally, and red dye was injected at 1 μL/min. The flow rate, influenced by gravity, filled the channel in 3.3 s. When oriented vertically and injected at the same rate, the channel filled in 2.1 s, 57.14% faster, as gravity’s force aligned with the flow direction (Figure 5c). Applying the same method to the serpentine channel, the horizontal orientation took 14 s to fill, while the vertical orientation filled in 13.1 s. The filling time increased by only 6.87%, demonstrating that the serpentine channel design not only prolongs collection duration but also mitigates the influence of wearer motion (Figure 5c). This illustrates the serpentine design’s effectiveness in extending sweat collection times while reducing the impact of the wearer’s movements on the collection process.

## 3. Sweat Detection by Microfluidic Sweat Glucose Monitoring Patch

### 3.1. Calibration of Standard Reference Curves for Sweat Sugar Concentration Assays

**Figure 6:**
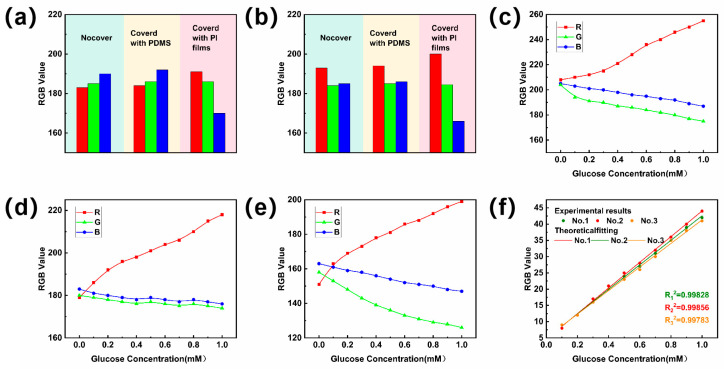
(**a**) RGB values of color-developing discs without color-developing reaction after covering the upper encapsulation layer. (**b**) RGB values of color-developing discs with color-developing reaction after covering the upper encapsulation layer. (**c**–**f**) RGB value when the color circle is illuminated by different kinds of light: (**c**) 2.5 W white light; (**d**) 5 W white light; (**e**) 3 W yellow light. (**f**) The standard reference curves were obtained by fitting the Δ*R* values of different concentrations of glucose solutions under different light (No. 1:2.5 W white light, No. 2:5 W white light, No. 3:3 W yellow light) to the colorimetric discs.

Given that the sensing layer of the microfluidic patch comprises a color-developing disc, the precision of the color values directly influences the accuracy of colorimetric detection. A comparative experiment was performed between a colorimetric disc without a color development reaction (R = 183, G = 185, B = 190) and one with a color development reaction (R = 193, G = 194, B = 199). As illustrated in Figure 6a,b, PDMS exhibits superior light transmittance compared to transparent PI film, making it a more suitable encapsulation material for colorimetric sensors. 

To quantify the glucose levels detected in the test, a standard reference curve was established at a room temperature of 25 °C. Initially, glucose solutions of varying concentrations (0.1 mM to 1 mM) were applied to the prepared colorimetric discs. Once the color development discs displayed full color, photographs were captured under 2.5 W white light with an FDRAX60 camera. The most dominant color block was identified using Color Hunter, and a color picker tool was used to obtain the R (red), G (green), and B (blue) values of this color block. Color-developed discs without glucose solution drops served as the standard color reference. Similarly, the initial color values under 2.5 W white light were recorded: R’ value, G’ value, and B’ value (Figure 6c). To minimize the light’s influence on measurement outcomes, the color development circle was imaged under both 5 W white light (Figure 6d) and 3 W yellow light (Figure 6e), yielding three sets of R values under varying lighting conditions. The three sets of R values were compared to the R’ values to calculate the Δ*R* values. Likewise, the differences between G and G’, and B and B’, were calculated as the Δ*G* and Δ*B* values, respectively. Across the three curves Δ*R*, Δ*G*, and Δ*B*, the Δ*R* values demonstrated a consistent trend and good fit, suggesting a linear correlation with glucose concentration. This allows the Δ*R* values to serve as a standard reference curve for colorimetric analysis (Figure 6f). To minimize data errors from color extraction, each color extraction was replicated six times. Through this methodology, glucose levels in sweat were accurately quantified, providing a robust approach for long-term glucose monitoring in sweat.

### 3.2. Microfluidic Sweat Patch Detects Sweat Sugar in the Human Body

**Figure 7:**
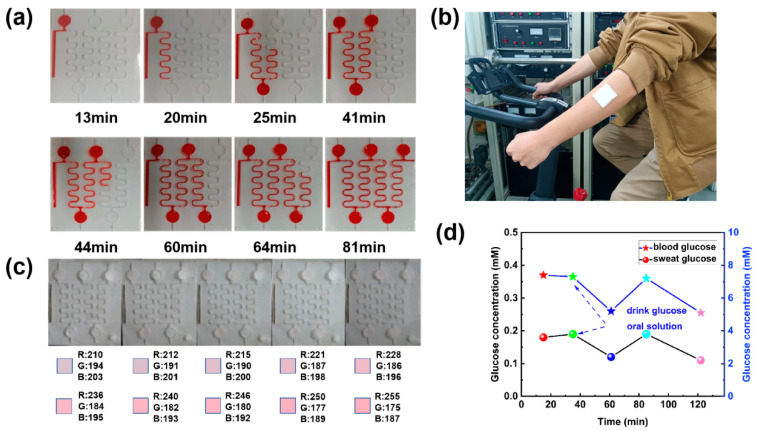
(**a**) In vitro simulation of the flow path and collection duration of red dye injected with a syringe pump. (**b**) An optical view of a microfluidic sweat glucose monitoring patch applied to a volunteer’s arm. (**c**) Color-rendering disc demonstrating sweat glucose detection in volunteers wearing microfluidic sweat glucose monitoring patches. (**d**) Dynamic monitoring results of sweat sugar.

To assess the microfluidic patch’s ability to collect and store sweat as anticipated, an in vitro simulation experiment was conducted. Circular holes were cut into a white plywood board to mimic sweat glands, and the microfluidic sweat patch was affixed such that these holes aligned with the patch’s sweat collection inlet. A syringe, connected via a catheter to the channel behind the board, simulated sweat collection at an injection rate of 1 μL/min. Experimental findings revealed that at a liquid flow rate of 1 μL/min, filling a single collection cell took approximately 20 min, while filling the entire microfluidic patch required about 1 h and 22 min (Figure 7a). The 2D diverter valve effectively redirected the flow, facilitating time-sequential collection. The patch was fully encapsulated, preventing any liquid spillage from the air valve or between adjacent structural layers, demonstrating the patch’s capability for efficient and contained sweat collection.

To further evaluate the actual detection efficacy of the microfluidic sweat glucose monitoring patch, the sweat samples were collected and analyzed by volunteers. Initially, volunteers cleaned their arm skin to eliminate dust and grease, preventing impurities from affecting the test results or detaching during collection. The microfluidic sweat monitoring patch was applied to the volunteer’s arm, and cycling exercise ensued at a room temperature of 30 °C (Figure 7b). To mitigate dehydration and excessive sweating due to the prolonged collection process, volunteers were instructed to consume significant amounts of water throughout the test. The first collection cell filled with sweat at 14 min and 54 s, prompting a color change in the color development disk. This change was compared to a standard colorimetric card, yielding Δ*R*_1_ = 13. Subsequently, volunteers consumed an oral dextrose solution to replenish glucose levels. Following the filling of each collection pool, blood samples were taken from volunteers to measure glucose levels (Figure 7c). The prolonged high-intensity exercise led to glycogen depletion, decreasing glucose concentration. After consuming a glucose oral solution for half an hour, sweat sugar concentration significantly increased, reflecting the body’s glucose absorption time from the ingested solution. Concurrently, the trend in sweat sugar changes mirrored those in blood glucose levels. Initially, sweat collection was rapid due to increased sweating from exercise onset. However, as exercise progressed, the reduced water content on the volunteers’ skin led to a slower sweating rate and collection pace (Figure 7d). The total collection time for the microfluidic sweat monitoring patch was approximately two hours, varying among individuals. Notably, this duration was significantly longer than previously achieved with such patches, indicating the patch’s efficacy in long-term sweat glucose monitoring (Table 1).

## 4. Conclusions

This paper introduces a microfluidic sweat monitoring patch designed for the long-term collection and glucose concentration detection of human sweat. Through innovative design, the patch’s usage is prolonged, facilitating the extended monitoring of glucose fluctuations. The patch comprises five collection units, four serpentine channels, and two distinct types of valves. In vitro simulated injection experiments confirmed the functionality of both the 3D gas valve and 2D diverter valve. Furthermore, appropriate plasma hydrophilic treatment, in conjunction with the serpentine channel design, effectively regulates the sweat flow rate. The experiment enables the detection of sweat glucose levels at two adjacent time intervals via the embedded color reaction on the color development disc. This approach offers insights into blood glucose fluctuations within the human body, demonstrating the patch’s potential for non-invasive, long-term glucose monitoring.

## Figures and Tables

**Table 1:** Performance comparisons of sweat glucose sensors.

Test Scope	Duration of the Test	Sweat/Blood Glucose Level Ranging	References
0.06–0.14 mM	20 min	0.001–0.02	[23]
0.06–0.8 mM	15 min	0.01–0.05	[24]
0.03–0.8 mM	60 min	0.01–0.02	[25]
0.01–1.11 mM	10 min	0.005–0.025	[26]
0–2 mM	25 min	0.01–0.11	[27]
0.01–1.11 mM	120 min	0.02–0.04	Our work

## Data Availability

The data presented in this study are available on request from the corresponding author.
